# Hard carbon spheres prepared by a modified Stöber method as anode material for high-performance potassium-ion batteries†

**DOI:** 10.1039/d1ra01488a

**Published:** 2021-04-21

**Authors:** Chenyang Fan, Mingyang Ou, Peng Wei, Jia Xu, Shixiong Sun, Yi Liu, Yue Xu, Chun Fang, Qing Li, Jiantao Han

**Affiliations:** State Key Laboratory of Material Processing and Die &Mould Technology School of Materials Science and Engineering, Huazhong University of Science and Technology Wuhan 430074 P. R. China jthan@hust.edu.cn

## Abstract

The Stöber method is a highly efficient synthesis strategy for homogeneous monodisperse polymer colloidal spheres and carbon spheres. This work delivers an extended Stöber method and investigates the synthesis process. By calcining the precursor under appropriate conditions, solid secondary particles of amorphous carbon (SSAC) and hollow secondary particles of graphitized carbon (HSGC) can be directly synthesized. The two materials have a nano-primary particle structure and a closely-packed sub-micron secondary particle structure, which can be used in energy storage. We find that SSAC and HSGC have high potassium-ion storage capacity with reversible capacities of 274 mA h g^−1^ and 283 mA h g^−1^ at 20 mA g^−1^ respectively. Significantly, SSAC has better rate performance with a specific capacity of 107 mA h g^−1^ at 1 A g^−1^.

## Abstract 2

A modified Stöber method synthesizes resorcinol-formaldehyde resin-based secondary particle hard carbon spheres as anode material for high-performance potassium-ion batteries.

## Graphical abstract

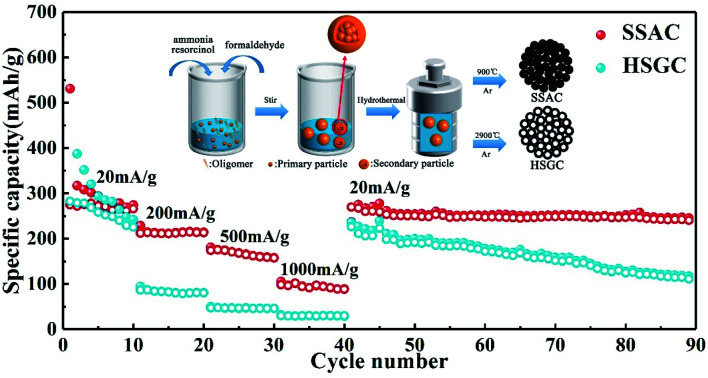

## 1. Introduction

In recent years, potassium-ion batteries (PIB), as a potential substitute for lithium-ion batteries, have received more and more attention from researchers. The main advantage of potassium ion batteries is that potassium is an element with abundant reserves, the price is much lower than that of lithium, its standard redox potential is lower than that of sodium, and the ion conductivity of potassium ions in the electrolyte is higher than that of lithium ions and sodium ions. Potassium ions with faster migration can bring better rate performance to potassium ion batteries.^1^ Carbon-based materials are the most promising materials for commercial anodes of potassium-ion batteries due to their low cost and wide resources. However, for the carbon-based anode of potassium ion batteries, the cycle stability and rate performance still need to be improved, which is inseparable from the structural design of carbon-based materials using suitable modification methods.^2–4^

The Stöber method is a highly efficient synthesis strategy for homogeneous monodisperse polymer colloidal spheres and carbon spheres. Carbon spheres synthesized by the Stöber method have excellent characteristics of carbon materials (such as good electronic conductivity, large pore distribution, high thermal and chemical stability) and great spherical morphology (good fluidity, low diffusion distance, and homogeneous geometry). In addition, carbon-based materials with various characteristics can be obtained through structural design,^5^ such as porous carbon,^6^ mesoporous carbon,^7^ hollow carbon,^8^ amorphous carbon,^9^ graphitized carbon,^10^*etc.* Carbon spherical particles synthesized by Stöber method have many applications in the field of electrochemical energy storage. Zou *et al.* designed an extended Stöber method. Hollow porous carbon spheres were made by microwave treatment and template method. The specific capacity of lithium-ion battery was 862 mA h g^−1^ at a current density of 0.1 A g^−1^.^11^ Jin *et al.* prepared resorcinol formaldehyde resin colloidal spheres with the different degrees of crosslinking, by changing the synthetic solvent. After further carbonization, a variety of hard carbon materials with certain structural differences were gained, and a three-phase model of hard carbon sodium storage was proposed subsequently.^12^ Zhang *et al.* reported a porous carbon material with egg-yolk shell structure. By tuning the co-sol–gel process of Stöber method, silica spontaneously constructed sacrificial template layer of the particles. The porous carbon had a potassium-ion storage capacity of 314 and 121 mA h g^−1^ at 50 and 5000 mA g^−1^, respectively.^13^

However, in the process of the expanded Stöber method to synthesize polymer colloidal spheres, researchers pay more attention to the control of the synthesis scheme of resorcinol-formaldehyde resin colloidal spheres,^14–16^ and less study the particle nucleation and growth processes in it. This may be due to (1) the Stöber synthesis process is too fast, and it is difficult to observe the process of primary particles growing into colloidal particles;^17^ (2) different from the Stöber method to synthesize colloidal silica spheres, the expanded Stöber synthesis process includes a subsequent aging process, which is in good agreement with the improved monomer addition model.^18^ Liu *et al.* explained the process of the expanded Stöber method to synthesize polymer colloidal spheres: formaldehyde and resorcinol undergo an addition polymerization reaction under the catalysis of ammonia to form long chains of dendritic oligomers, and then form polymer clusters, which collapse to form primary particles. Because of the large amount of ammonia ion adsorbed on the surface of primary particles, the particles are stable. The subsequent growth of the primary particles undergoes a sol–gel process and an aging process. Monomers, oligomers, and polymer clusters continue to aggregate and react on the surface of the particles, and finally grow into colloidal spheres.^19^

Here we report an expanded Stöber synthesis method, by mixing resorcinol with ammonia, and adding the mixed solution and formaldehyde solution to deionized water, then obtaining modified resorcinol formaldehyde resin after stirring and hydrothermal process. We speculate that the expanded Stöber method is more similar to the controlled aggregation-based model in the classical Stöber of silica.^20^ Finally, SSAC and HSGC are obtained after calcining at the different temperatures. SSAC and HSGC are densely packed with amorphous nanoparticles and hollow graphitized nanoparticles, and have good potassium-ion storage performance, with a high reversible capacity of 274 mA h g^−1^ and 283 mA h g^−1^ at a current density of 20 mA g^−1^, respectively. SSAC has more abundant defects and porous structure. The 10 nm-size amorphous primary particles provide a very short potassium-ion diffusion distance, ensuring that SSAC has good rate performance and still has a reversible capacity of 107 mA h g^−1^ at 1 A g^−1^. Although HSGC has a hollow primary particle structure, the highly graphitized particle edges are not conducive to the diffusion of potassium ions, the rate performance is slightly poor.

## 2. Experimental

### 2.1 Material synthesis

#### 2.1.1 Synthesis of resorcinol formaldehyde resin precursor

1 ml of 85 wt% ammonia and 1.4 g of resorcinol was added to 20 ml of DI water to obtain solution A. And 0.7 g 37 wt% formaldehyde was added to 20 ml deionized water to obtain solution B. Then, the solutions A and B were slowly dropped into 40 ml of DI water at the same time and stirred for 12 h to obtain the precursor solution RF-RT at room temperature. Then RF-RT was added to a sealed reactor and heated at 100 °C for 24 hours and the reaction product was washed by centrifugation using ethanol and deionized water, finally dried to obtain the precursor RF-RT-24 h. Similarly, the same experiment was carried out to obtain RF-LT and RF-LT-24 h at 0 °C. As a control group, the ammonia water was replaced with the same ratio of Na_2_CO_3_, and the precursor solution obtained by stirring at room temperature was spray-dried to prepare spray drying-RF.

#### 2.1.2 Synthesis of hard carbon spheres

The precursor RF-RT-24 h and spray drying-RF were put into a graphite crucible and heated at 900 °C for 2 hours at a heating rate of 2 °C per minute in a flowing Ar atmosphere to obtain SSAC and spray drying-HC. Similarly, RF-RT-24 h was heated at 2900 °C to obtain HSGC.

### 2.2 Materials characterization

An X-ray diffractometer (Empyrean Nano, PANALYTICAL, The Netherlands) was used to collect XRD patterns with Cu-K_α_ radiation at a scan rate of 12° min^−1^, and X-ray total scattering experiments were performed on it too with Ag Kα radiation to get pair distribution function (PDF) patterns. X-ray absorption near edge structure (XANES) was conducted at BL12B beamline in the National Synchrotron Radiation Laboratory (BSRL) in Hefei. Fourier-transform infrared spectroscopy (FT-IR) was performed on a Bruker Vector 22 spectrometer. Raman spectra were obtained by a Raman spectrometer using an Ar ion laser with a wavelength of 532 nm (LabRAM HR800, Horiba, France). The morphology of the material was recorded by a field emission scanning electron microscope (VEGA 3 SBH, TESCAN, Czech Republic). TEM was measured using a transmission electron microscope (Tecnai G2 F30, FEI, USA), and used cryostat microtome (Leica UC7FC7) and FIB microtome (FEI Helios Nanolab 600i) to thin the sample. The BET surface area data was gained for nitrogen adsorption at 77 K on the Tristar II3020 instrument.

### 2.3 Electrochemical measurements

The electrode was prepared by mixing 80 wt% of SSAC or HSGC, 10 wt% of the conductive agent (Super-P), and 10 wt% of polymer binder (CMC : SBR = 1 : 1) uniformly into a slurry, and applied it on the copper foil with thickness of 300 μm, and then dried in a vacuum oven at 100 °C for 12 hours. We assembled the CR2032 coin-type cells in a glove box filled with Ar atmosphere, using K metal as the counter electrode, glass fiber membrane as the separator, and 0.8 M KPF6 in EC and DEC (1 : 1, v/v) for the electrolyte. Galvanostatic charge and discharge tests was performed on the land battery test system (LAND 2001 CT, China), including cycle tests and rate tests. The cyclic voltammetry (CV) tests were carried out on the Chenhua electrochemical workstation (CHI760E, China), the scanning speed is 0.1 mV s^−1^. In the galvanostatic intermittent titration technique tests (GITT), each single GITT titration was charged or discharged at a current density of 50 mA g^−1^ for 15 minutes, followed by a 30 minutes' relaxation. All electrochemical tests were carried out at room temperature, and the voltage range of the potassium ion battery was 2.0–0.01 V *versus* K/K^+^.

## 3. Results and discussion

### 3.1 Structure analysis and morphology

**Fig. 1:**
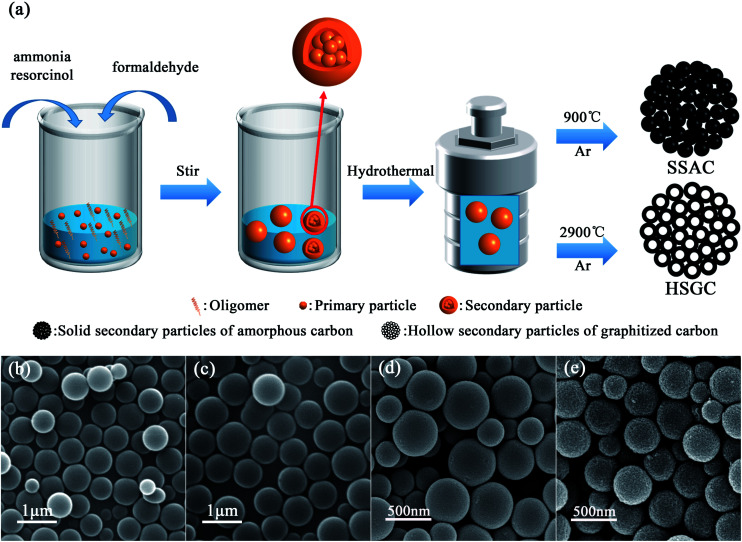
(a) Schematic illustration of the process of synthesizing resorcinol-formaldehyde resin colloidal spheres (RF spheres) by Stöber method, and its carbonized products SSAC and HSGC. The SEM images of (b) RF-RT, (c) RF-RT-24 h, (d) SSAC and (e) HSGC.

**Fig. 2:**
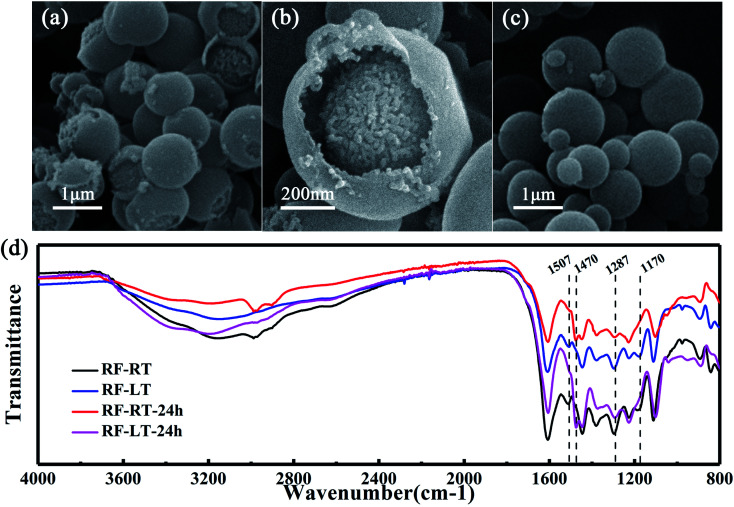
The images recorded that low-temperature conditions slowed down the Stöber synthesis process. The SEM images of (a and b) RF-LT and (c) RF-LT-24 h. (d) FTIR spectra of colloidal spheres synthesized by Stöber method at room temperature and low temperature.

We have made some modifications to the Stöber process. The schematic diagram of the preparation of SSAC and HSGC is shown in Fig. 1a. We fully learn from the experience of researchers on the synthesis of colloidal silica spheres by the Stöber method. Han *et al.* found that at low ammonia concentrations, as the reaction time increases, oligomers more spontaneously grow into new primary particles, and less aggregate and grow on the surface of original primary particles.^21^ By adding the mixed solution of resorcinol and ammonia water slowly and dropwise to the deionized water at the same time as the formaldehyde solution, the monomer concentration and ammonia concentration in the reaction system are controlled at an extremely low level. Therefore, the surface of the primary particles in the modified Stöber process cannot adsorb enough ammonia ions, which makes them more unstable and easier to aggregate. In addition, the monomer concentration in the system is extremely low, and the primary particles are difficult to grow. Therefore, with the gradual addition of monomer and ammonia, secondary particle colloidal spheres will be formed, assembled by a large number of primary particles. The details of the reaction process have been presented in Fig. 2.

We performed the modified Stöber process at room temperature, after adding resorcinol, formaldehyde, and ammonia, the digital photograph of the solution is shown in Fig. S1a,† rapidly changing from colorless to milky white, then stirring for 12 hours to obtain RF-RT, corresponding SEM image is shown in Fig. 1b, which are spherical particles with a smooth surface and a size range of 300–700 nm. And the RF-RT-24 h was gained after hydrothermal aging as shown in Fig. 1c, which particle size is larger than that of RF-RT, the solution is brownish red in Fig. S1c,† indicating that the particles had undergone a growth process. Fig. 1d and e are the SEM images of SSAC and HSGC obtained by carbonization of the precursor RF-RT-24 h at 900 °C and 2900 °C in an argon atmosphere, respectively. Both SSAC and HSGC are spherical carbon particles with a size range of 200–500 nm. SSAC has a smooth surface, while HSGC has a very rough surface, indicating that as the carbonization temperature increases, SSAC and HSGC have a significant structural difference.

To verify the mechanism of our modified Stöber synthesis process, we carried out the reaction at a low temperature of 0 °C. Under this condition, the reaction rate of the modified Stöber process is reduced to a lower level, making it easier for us to observe the details of particle growth. Fig. 2a and b are the SEM images of RF-LT obtained by stirring for 12 hours after adding resorcinol, formaldehyde, and ammonia at 0 °C. The morphology of RF-LT is different from RF-RT, many RF spheres that are not completely enclosed can be observed, which shape is like a “pomegranate”, the inside is made up of many small primary particles below 10 nm, the outside is a layer of wall structure, the inside of the wall adheres to the primary particles, and the outside of the wall is as smooth as the RF spheres of RF-RT. Such a “pomegranate” structure of RF spheres can very intuitively prove its formation process a large number of primary particles assembled into colloidal spheres of secondary particles. After 24 h of the hydrothermal aging process, RF-LT-24 h is obtained, as shown in Fig. 2c. At this time, the RF spheres become complete smooth particles again, with a size range of 300–1000 nm, indicating that the hydrothermal aging process causes the RF-LT to grow again, and the RF spheres with such a “pomegranate” structure are the intermediate of the modified Stöber process.

To further prove that the modified Stöber processes are consistent at 0 °C and room temperature, we measured Fourier transform infrared spectra for these four kinds of RF spheres, as displayed in Fig. 2d. RF-RT and RF-LT have almost the same peak position and peak shape, RF-RT-24 h and RF-LT-24 h also have almost the same peak position and peak shape, indicating that our prediction is correct. The difference between RF-RT and RF-RT-24 h is the same as the difference between RF-LT and RF-LT-24 h. This difference proves that as the hydrothermal reaction and aging process proceeds, the degree of cross-linking inside the RF spheres continues to rise. The bands located at 1507 cm^−1^ and 1170 cm^−1^ for RF-RT and RF-LT are attributed to the –C=C stretching vibration of the benzene ring in resorcinol and the –C–O stretching vibration on the benzene ring that undergoes orthogonal addition, it shows residual reaction monomers and oligomers. RF-RT-24 h and RF-LT-24 h have a band at 1470 cm^−1^, which is attributed to the –C–H vibration on –CH_2_– and –O–CH_2_– connected between oligomers, indicating that the degree of crosslinking in the system further increases with hydrothermal reaction and aging, and the band at 1287 cm^−1^ becomes weaker, which proves that the ether connection between oligomers is reduced, and it also confirms that the degree of crosslinking in the system increases.^22–24^

**Fig. 3:**
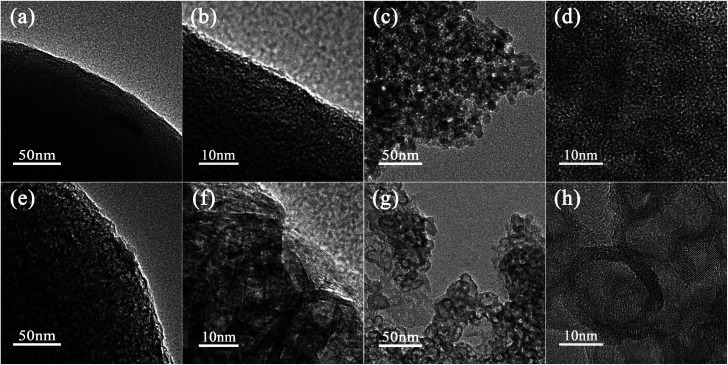
The TEM image of (a and b) SSAC and (e and f) HSGC. The TEM image of (c and d) SSAC after FIB fragmentation and (g and h) HSGC after FIB fragmentation.

To further characterize the microstructure of SSAC and HSGC carbonized by RF-RT-24 h, we performed TEM characterization. Fig. 3a, b, e and f are TEM images of SSAC and HSGC, respectively. It can be observed that SSAC is a highly amorphous structure, and no carbon layer structure can be seen inside the particles, and it is a dense large particle, while HSGC particles are highly hollow and graphitized, and a large number of carbon layers can be seen connected, and the interlamellar spacing *d* (002) is 0.338 nm. It is difficult to distinguish the internal structure of SSAC and HSGC. Fig. S2a and b† are TEM images of HSGC after embedding frozen sections, and the information obtained is the same as that of Fig. 3e and f.

To analyze the microstructure of SSAC and HSGC more deeply, we performed FIB fragmentation on them. Fig. 3c, d, g, and h are TEM images of SSAC and HSGC after FIB fragmentation, respectively. At this time, more internal structures of particles are exposed, and the morphology of SSAC becomes a large number of amorphous nanoparticles below 10 nm, while HSGC exposes a large number of hollow graphitized nanoparticles of 10–20 nm. The generation of primary nanoparticles in SSAC and HSGC is derived from the polymer primary particle structure that exists in the precursor itself. After the carbonization process at the different temperatures, the unique “pomegranate” structure of the precursor is maintained, resulting in such unique solid secondary particles of amorphous carbon (SSAC) and hollow secondary particles of graphitized carbon (HSGC).

**Fig. 4:**
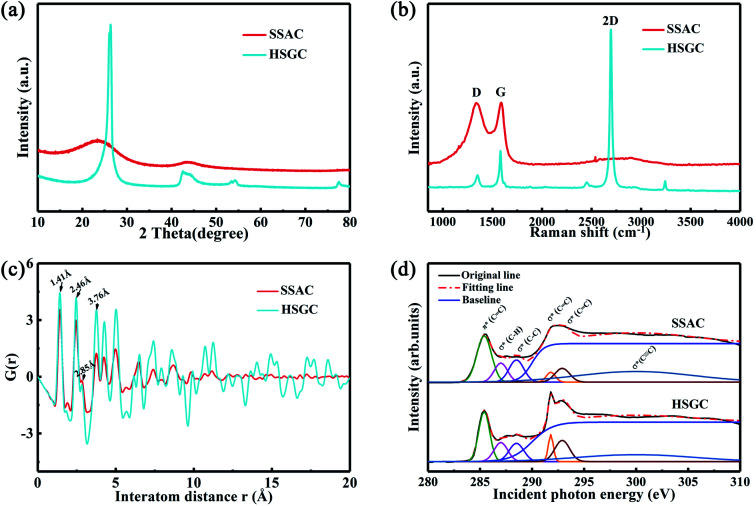
Structure characterization of SSAC and HSGC. (a) XRD patterns of SSAC and HSGC, (b) Raman spectra patterns of SSAC and HSGC, (c) PDF patterns of SSAC and HSGC, (d) fitted XANES curves of SSAC and HSGC.

The X-ray diffraction pattern of Fig. 4a shows the structural difference between SSAC and HSGC. SSAC has two broad peaks at 23.5° and 43.3°, representing (002) and (100) crystal planes, calculated from the Bragg formula:*λ* = 2*d* sin *θ*,*d*(002) is 0.3858 nm, indicating that SSAC is a typical amorphous carbon structure, which suggests a lot of defects and amorphous structure; The diffraction peak of (002) crystal plane of HSGC is located at 26.2°, *d*(002) is 0.3381 nm, which is very close to the interlayer spacing of graphite, and the peak intensity is stronger and the peak width is narrower, which shows that the degree of graphitization of HSGC is greatly increased and defects are greatly reduced. The Raman scattering spectrum is shown in Fig. 4b and Table 1, SSAC shows wider D band and G band, after fitting, as shown in Fig. S4,† the ratio of *I*_G_/*I*_D_ is 0.2570, calculate *L*_a_ of carbon materials by this formula:^25^*L*_a_ = (2.4 × 10−^10^)*λ*_nm_^4^(*I*_G_/*I*_D_)where *λ*_nm_ is the laser wavelength of the Raman spectrometer at 532 nm, and the calculated *L*_a_ value is 4.9540 nm, indicating that the SSAC has high defects and poor carbon layer development. And HSGC has a narrower D band and G band, the ratio of *I*_G_/*I*_D_ is 1.9229, which is much larger than SSAC, the *L*_a_ value is 37.0064 nm indicating its higher degree of graphitization and defects. Very unique is that HSGC has a very high *I*_2D_/*I*_G_ value of 3.7426, and this high ratio is often found in graphene materials.^26,27^ And the BET data of SSAC and HSGC are also recorded in Table 1 and Fig. S3,† SSAC has a very large specific surface area of 301.2237 m^2^ g^−1^ and a large pore volume, while specific surface area of HSGC is only 12.0221 m^2^ g^−1^. This is due to the high-temperature carbonization process that causes the pores in the carbon material to disappear and close.

**Table 1:** Structure parameters of SSAC and HSGC

	*d*(002) (nm)	*I*_G_/*I*_D_	*I*_2D_/*I*_G_	*L*_a_ (nm)	% sp^2^	BET (m^2^ g^−1^)
SSAC	0.3858	0.2570	—	4.9540	54.85	301.2237
HSGC	0.3381	1.9229	3.7426	37.0064	99.29	12.0221

As shown in Fig. 4c, the PDF signal strength trend of HSGC decays slowly, while the SSAC decays faster, indicating that the carbon layer of HSGC is larger and better developed than that of SSAC, which is consistent with the Raman data. Since the peak area of PDF represents the coordination number, the smaller integrated area of the SSAC peak means that the coordination number of SSAC is less than that of HSGC, indicating that SSAC has more defects.^28–30^ We also performed XANES measurement and fitted the obtained data, the *σ*(C=C) peak area and peak intensity of HSGC are larger than those of SSAC, and are similar to the *σ*(C=C) peak of graphite in Fig. S5,† indicating that the carbon layer of HSGC is better developed and more carbon six-membered rings are formed. And we further analyzed the proportion of sp^2^ hybrid carbon atoms of the two carbon materials, the calculation formula is as follows:^30^*S*_σ(all)_ = *S*_σ(C=C)_ + *S*_σ(C–H)_ + *S*_σ(C–C)_ + *S*_σ(C≡C)_
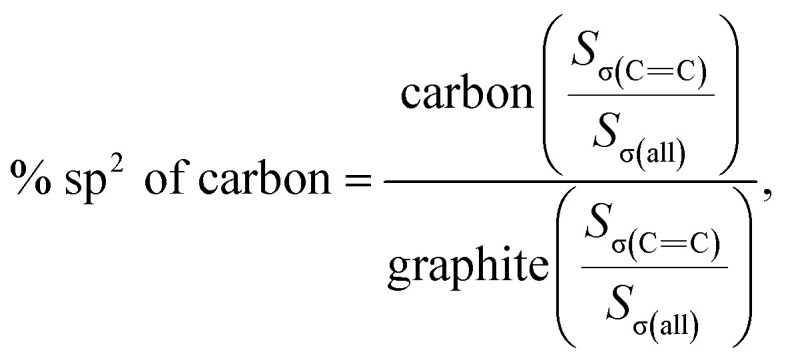
where *S* represents the integral area of the peak, the proportion of carbon atoms hybridized by sp^2^ of graphite is recorded as 100%, and % sp^2^ is the proportion of carbon atoms hybridized by sp^2^ of the sample relative to the graphite, the calculated % sp^2^ ratio of HSGC is 99.29%, which is very similar to graphite. The % sp^2^ ratio of SSAC is only 54.85%, reflecting that SSAC has a large number of amorphous structures and defects.^31–33^ From the elemental analysis results in Table S1,† it can be seen that SSAC contains a large amount of non-carbon elements, while HSGC is almost pure carbon, which is due to the element loss caused by the high-temperature carbonization process.

### 3.2 Electrochemical performance

**Fig. 5:**
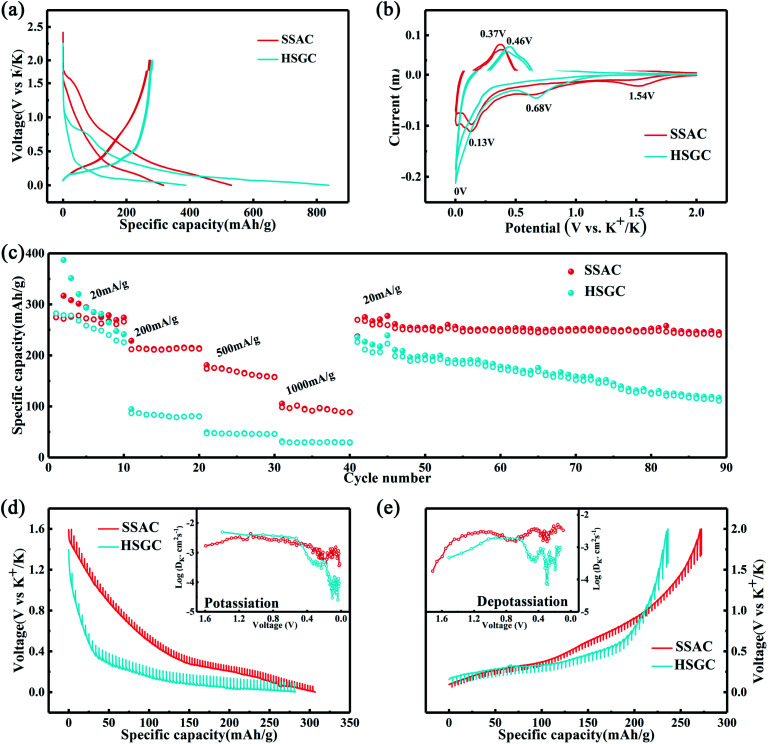
SSAC and HSGC potassium ion battery performance test. (a) Discharge and charge curves of the first two cycles of SSAC and HSGC electrodes. (b) CV curves of the first two cycles of SSAC and HSGC electrodes. (c) Rate performance of SSAC and HSGC electrodes at different rates, followed by cycling performance (d and e) GITT profiles and diffusion coefficients of SSAC and HSGC.

Then we assembled potassium-ion half-cells to evaluate the potassium-ion storage behavior of SSAC and HSGC. The galvanostatic charge/discharge curves and cyclic voltammetry curves of the first two cycles are revealed in Fig. 5a and b. The first cycle charge/discharge capacity of SSAC and HSGC are 274/531 mA h g^−1^ and 283/838 mA h g^−1^ at a current density of 20 mA g^−1^ between the voltage range of 2.0–0.01 V (*vs.* K/K^+^), and the second cycle are 271/317 mA h g^−1^ and 279/387 mA h g^−1^. The charge–discharge curve of SSAC has a high platform and slope type, and the specific capacity is mostly concentrated below 1 V, similar to a typical defective carbon.^34^ The HSGC has a low charge–discharge curve and a long plateau, the specific capacity is mostly concentrated below 0.5 V, similar to typical graphite.^35^ The CV curve of SSAC and HSGC at 0.1 mV s^−1^ can correspond to the charge and discharge curve, the broad cathodic peaks of SSAC around 1.54 V/0.68 V and HSGC around 0.68 V in the first cycle disappeared in the second cycle, which could be due to the formation of solid electrolyte interface layer, the trapping of potassium ions at defects and the slow decomposition of electrolyte.^36,37^ The cathodic peaks of SSAC and HSGC at about 0.15 V and 0 V correspond to the insertion of potassium ions into the carbon layer, while the anode peaks at about 0.37 V and 0.46 V correspond to the extraction of potassium ions from the carbon layer.^38^ The difference is that the cathode peak of HSGC is biased towards low voltage, which corresponds to the galvanostatic cycle curve. The capacity of SSAC and HSGC at different rates is shown in Fig. 5c, SSAC has good rate performance, exhibiting discharge capacity of 316, 225, 182, 109 mA h g^−1^ at 20, 200, 500, 1000 mA g^−1^, respectively, and after 90 cycles of charge–discharge test, the capacity retention rate is still 80%. The SEM image of the SSAC electrode before and after the cycle is shown in Fig. S7.† It can be observed that a large number of SSAC particles are accumulated on the original electrode before the cycle, and the electrode after the cycle is covered with a smooth and complete solid electrolyte interface layer, which proves the cycle stability of the SSAC electrode. HSGC has a slightly worse electrochemical performance, exhibiting discharge capacities of 387, 96, 53, 34 mA h g^−1^ at 20, 200, 500, and 1000 mA g^−1^, and the final capacity retention rate is 31%. This result is because SSAC has a wide *d*(002) layer spacing of 0.3858 nm, and the carbon layer is short and rich in defects, which facilitates the transmission and storage of potassium ions. While HSGC has a highly hollow structure and a short potassium-ion transmission path, but the highly graphitized carbon layer has a narrow *d*(002) layer spacing, which is not conducive to the diffusion of large-scale potassium ions. We also prepared carbon spheres without secondary structure through spray drying method to compare with SSAC, and performed electrochemical performance as shown in Fig. S8,† exhibiting discharge capacity of 282, 83, 24, 4 mA h g^−1^ at 20, 200, 500, 1000 mA g^−1^, and the final capacity retention rate is 67%, indicating that the secondary structure will improve the cycle and rate performance of electrode materials.^39–41^

To verify our above conjecture and explore the kinetic process of potassium-ion storage, we applied the galvanostatic intermittent titration technique (GITT) to calculate the diffusion coefficients (*D*_k_) of SSAC and HSGC electrodes at different voltages, which is calculated by Fick's second law and the following equation:^37,42^
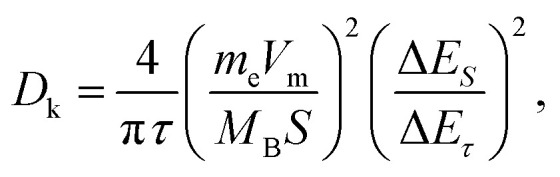
in the equation, *m*_B_, *M*_B_, and *V*_m_ represent the mass, molar mass, and molar volume of SSAC and HSGC, respectively. *τ* expresses the duration of the pulse current, and *S* represents the contact area for the electrode. Δ*E*_*S*_ expresses the quasi-equilibrium potential difference during the current pulse, and Δ*E*_*τ*_ expresses the potential difference before and after the current pulse, single GITT process is shown in Fig. S6.†Fig. 5 d and e show the potential response of the electrode during the GITT measurement, and the change of *D*_k_ value with voltage. The *D*_k_ values of SSAC and HSGC above 0.5 V are approximate during the discharge process, while the potassium ion diffusion coefficient of SSAC below 0.5 V is one to two orders of magnitude higher than HSGC. And under 0.5 V during charging, the *D*_k_ value of SSAC is also much higher than HSGC. This fully indicates that the SSAC with a large number of defects and large interlayer spacing has a higher potassium ion diffusion coefficient, and also proves that SSAC has the better electrochemical performance.

## 4. Conclusions

In summary, SSAC and HSGC are secondary particulate carbon spheres synthesized in one step by the modified Stöber method. The modified Stöber method controls the nucleation reaction in the Stöber synthesis process by regulating the monomer and ammonia concentration in the system, the primary particles are more likely to agglomerate at very low concentrations, so the resorcinol-formaldehyde resin colloidal spheres with secondary particle structure are designed, and the secondary particle carbon spheres are obtained by calcination under argon atmosphere. SSAC and HSGC have both an amorphous/hollow graphitized nano-primary particle structure and a closely packed sub-micron secondary particle structure, and showed good potassium ion storage capacity, with high reversible capacities of 274 mA h g^−1^ and 283 mA h g^−1^ at 20 mA g^−1^. SSAC has better rate performance and still has a specific capacity of 107 mA h g^−1^ at 1 A g^−1^. This work reveals that the Stöber method is an effective and scalable strategy for synthesizing carbon spheres with different microstructures, which is beneficial to improve the electrochemical performance of carbon anode materials for PIB as well as other electrochemical energy storage systems, and deepen the understanding of hard carbon structure.

## Conflicts of interest

There are no conflicts to declare.

## Supplementary Material

RA-011-D1RA01488A-s001
