# Supplementary material for: Hard carbon spheres prepared by a modified Stöber method as anode material for high-performance potassium-ion batteries
Source: RSC Adv. 2021 Apr 21;11(24):14883–90. doi: 10.1039/d1ra01488a (PMC8698061; doi:10.1039/d1ra01488a)
Supplement: RA-011-D1RA01488A-s001 [file RA-011-D1RA01488A-s001.pdf]

Electronic Supplementary Material (ESI) for RSC Advances.
This journal is © The Royal Society of Chemistry 2021

Supplementary Information

Hard carbon spheres as anode material for high-performance potassium-ion battery

Chenyang Fan‡, Mingyang Ou‡, Peng Wei, Jia Xu, Shixiong Sun, Yi Liu, Yue Xu, Chun Fang, Qing Li and Jiantao Han*

State Key Laboratory of Material Processing and Die &Mould Technology

School of Materials Science and Engineering

Huazhong University of Science and Technology

Wuhan 430074, P. R. China

Corresponding Author

*E-mail: jthan@hust.edu.cn (Prof. Jiantao Han)

‡ C. Fan and M. Ou contributed equally to this work.

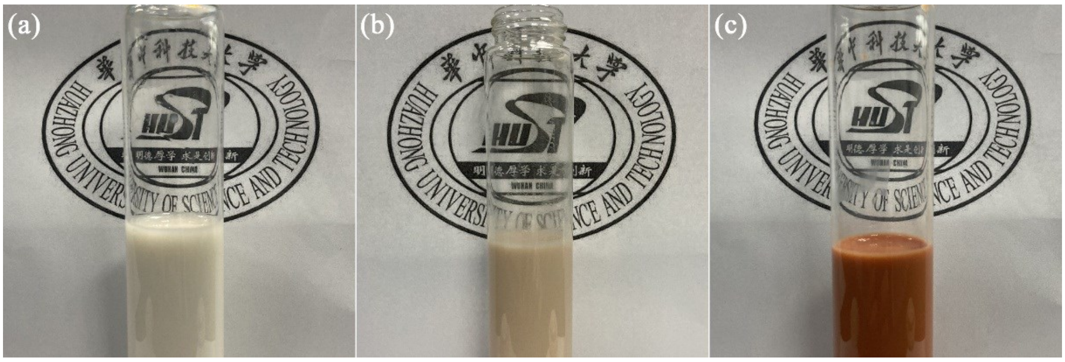

Fig. S1 The products at different stages of the Stöber synthesis process at room temperature, (a) after adding the reaction materials, (b) after 12 h stirring, and (c) after 24 h hydrothermal reaction.

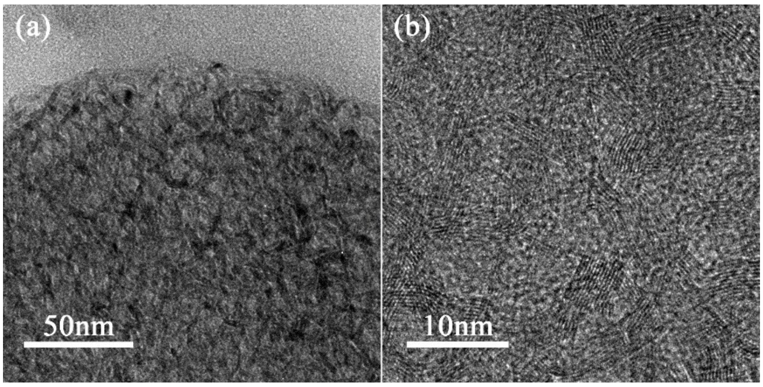

Fig. S2 The TEM images of (a, b) HSGC after embedding frozen sections.

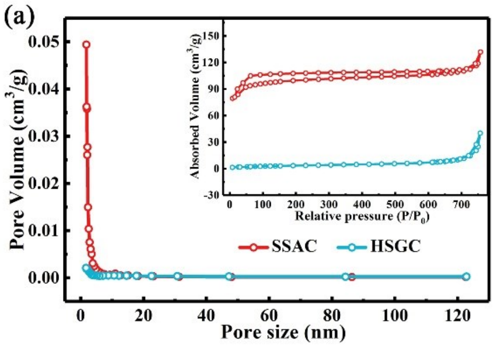

Fig. S3. (a) Nitrogen adsorption–desorption isotherm curves and pore size distribution of SSAC and HSGC.

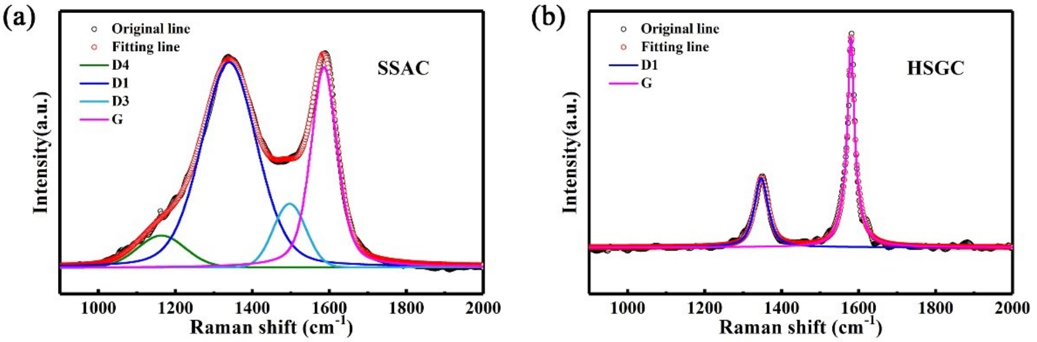

Fig. S4. (a, b) Fitted Raman spectra curves of SSAC and HSGC.

Table. S1 Elemental analysis results of SSAC and HSGC.

	C(%)	H(%)	O(%)	N(%)
SSAC	84.132	1.594	11.709	2.61
HSGC	98.092	0.152	0.806	0.95

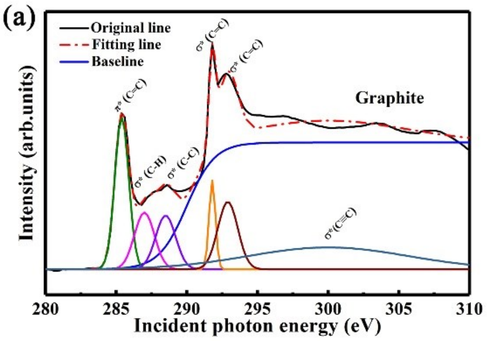

Fig. S5. (a) Fitted XANES curves of graphite. The graphite was purchased from BTR New Material Group Co., Ltd.

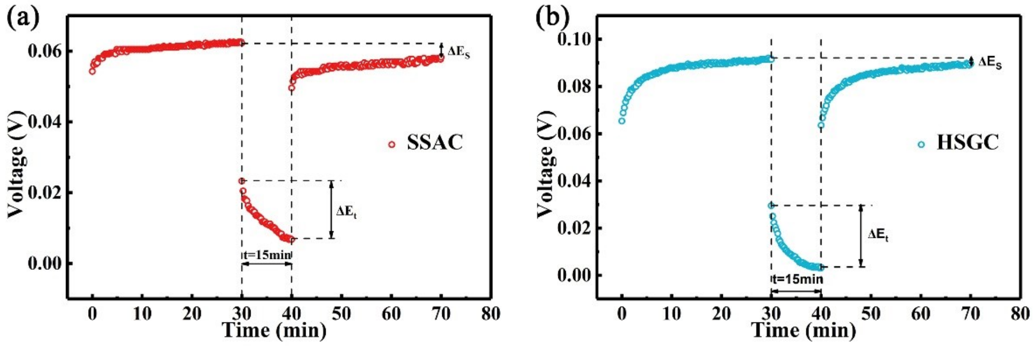

Fig. S6. (a, b) Time vs Voltage profiles for a single GITT titration of SSAC and HSGC.

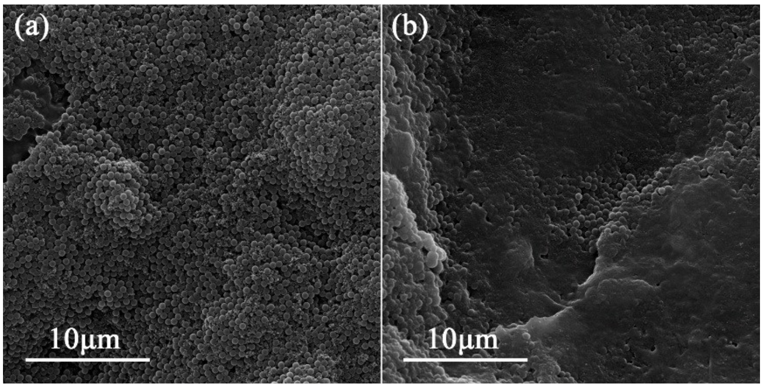

Fig. S7. The SEM images of (a, b) SSAC electrode before and after 90 cycles.

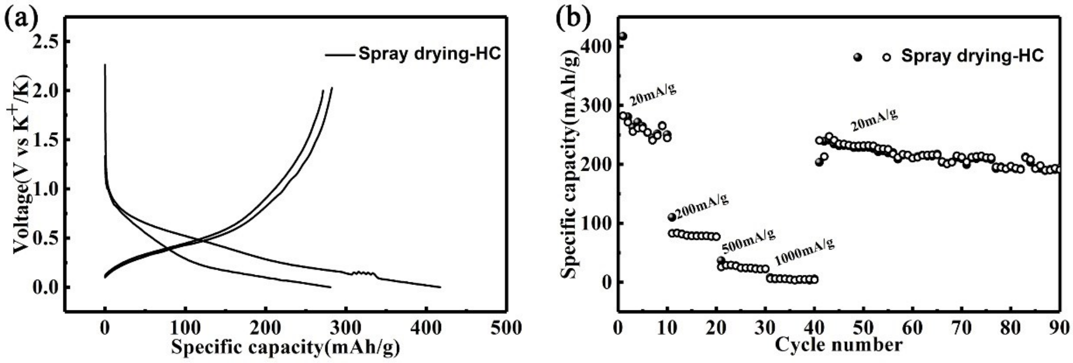

Fig. S8. (a) Discharge and charge curves of the first two cycles of Spray drying-HC electrodes at 20 mA/g. (b) Rate performance of Spray drying-HC electrodes at different rates, followed by cycling performance.

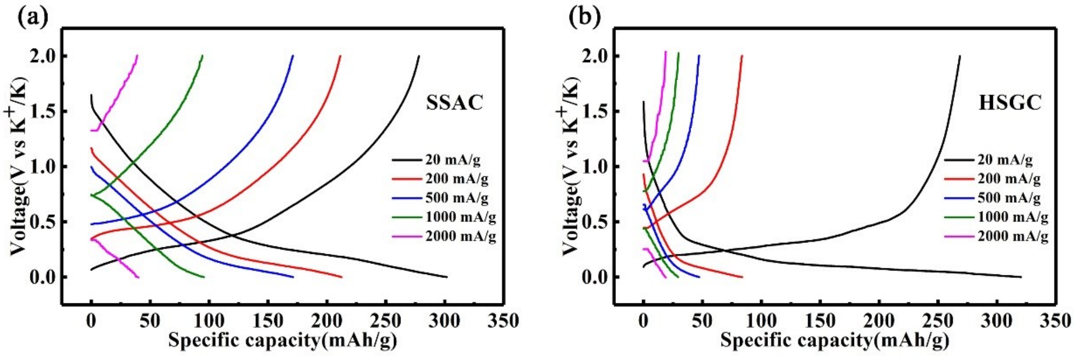

Fig. S9. (a, b) Discharge and charge curves of SSAC and HSGC at different current densities.
